# Impaired Voluntary Control in PTSD: Probing Self-Regulation of the ACC With Real-Time fMRI

**DOI:** 10.3389/fpsyt.2018.00219

**Published:** 2018-05-30

**Authors:** Jana Zweerings, Eliza M. Pflieger, Krystyna A. Mathiak, Mikhail Zvyagintsev, Anastasia Kacela, Guido Flatten, Klaus Mathiak

**Affiliations:** ^1^Department of Psychiatry, Psychotherapy and Psychosomatics, Medical School, RWTH Aachen University, Aachen, Germany; ^2^Brain Imaging Facility, Interdisciplinary Centre for Clinical Studies (IZKF), Medical Faculty, RWTH Aachen University, Aachen, Germany; ^3^Euregio-Institut für Psychosomatik und Psychotraumatologie, Aachen, Germany; ^4^JARA—Translational Brain Medicine, Aachen, Germany

**Keywords:** PTSD, self-regulation, emotion regulation, real-time fMRI, anterior cingulate cortex (ACC), neurofeedback

## Abstract

**Background:** Post-traumatic stress disorder (PTSD) is characterized by deficits in the self-regulation of cognitions and emotions. Neural networks of emotion regulation may exhibit reduced control mediated by the anterior cingulate cortex (ACC), contributing to aberrant limbic responses in PTSD.

**Methods:** Real-time fMRI neurofeedback (rt-fMRI NF) assessed self-regulation of the ACC in nine patients with PTSD after single trauma exposure and nine matched healthy controls. All participants were instructed to train ACC upregulation on three training days.

**Results:** Both groups achieved regulation, which was associated with wide-spread brain activation encompassing the ACC. Compared to the controls, regulation amplitude and learning rate was lower in patients, correlating with symptom severity. In addition, a frontopolar activation cluster was associated with self-regulation efforts in patients.

**Conclusions:** For the first time, we tested self-regulation of the ACC in patients with PTSD. The observed impairment supports models of ACC-mediated regulation deficits that may contribute to the psychopathology of PTSD. Controlled trials in a larger sample are needed to confirm our findings and to directly investigate whether training of central regulation mechanisms improves emotion regulation in PTSD.

## Introduction

Post-traumatic stress disorder (PTSD) is characterized by impaired self-regulation (1, 2). In particular, the regulation of emotional responses to a wide range of stimuli is dysfunctional. Whereas hyperarousal, i.e., the failure to downregulate arousal when no threat is imminent anymore, is often observed in PTSD, patients also exhibit excessive control yielding reduced arousal and dissociative symptoms (3). At the neural level, studies indicate hyperactivation of the amygdala and insula in several anxiety disorders, however, only PTSD was linked to hypoactivation of the dorsal and rostral anterior cingulate cortices (ACC) (4). Hence, the disorder may be associated with a functional impairment of the ACC that may contribute to deficits in self-regulation. However, neural mechanisms of self-regulation in PTSD remain elusive. To examine the direct linkage between PTSD and self-regulation ability of brain activation in the ACC, we applied real-time functional magnetic resonance imaging neurofeedback (rt-fMRI NF).

### Impaired self-regulation in PTSD

Symptoms of re-experiencing, avoidance, emotional numbing, and hyperarousal may emerge after an individual was exposed to a traumatic event. Frequently these symptoms subside; however, if they persist PTSD may be diagnosed. Impaired self-regulation has been proposed as a central mechanism of the disorder (1). This is supported by a link between PTSD and impulse regulation (5). Furthermore, a strong association between PTSD and deficits in emotion regulation is well established (6, 7). Functional imaging studies consistently point at a dysfunction in emotion regulation networks, involving deficits in the control of positive and negative affect (for a review, see (8)). From clinical and research perspective, impaired self-regulation may be central for PTSD whereas deficits in bottom-up processes may be particularly relevant in the context of other anxiety disorders (4).

Connectivity of the ACC with limbic structures such as the amygdala and its potential coupling with the hypothalamic-pituitary-adrenal (HPA) axis render the ACC central in PTSD research [for a review, see (9)]. Based on a comprehensive meta-analysis, Kohn et al. (10) consider the dorsal ACC a core node for emotion regulation. Accordingly, PTSD was associated with hypoactivation of the dorsal and rostral ACC in response to negative emotional stimuli while the amygdala and insula were hyperactive (4). Offringa et al. (11) reported reduced activation in the rostral ACC in an interference task with trauma-unrelated emotional stimuli indicating a generalized emotion regulation deficit. In a similar vein, patients with PTSD exhibited altered connectivity between the ACC and the amygdala during rest (12). A comprehensive meta-analysis found consistent hypoactivation in medial prefrontal regions and the dorsal ACC upon comparing patients with PTSD to trauma-exposed individuals who did not meet diagnostic criteria (7). Amygdala activation was increased in all trauma-exposed individuals pointing to a core role of the ACC in the psychopathology of PTSD. In line with this assumption, symptom reduction in PTSD has been associated with an increase in subgenual ACC activation, whereas the amygdala activation did not correlate with improvement (13). On a structural level, a meta-analysis showed volume reductions in bilateral ACC while amygdala volumes were not significantly reduced in PTSD relative to trauma-exposed controls (14). Moreover, antidepressant treatment increased phase coherence between low-frequency blood oxygen level-dependent (BOLD) fluctuations in the ACC and limbic regions, medial thalamus, pallidostriatum, and amygdala, which may reflect improved emotion regulation in depression (15). Conceivably, the ACC is instrumental in self-regulation. In particular, its dysfunction may mediate regulation deficits in PTSD and subsequent symptomatology.

### Self-regulation of brain activity

Rt-fMRI NF yields the unique advantage to study self-regulation of brain activation in a circumscribed target region directly (16–21). In comparison to psychopharmacological treatments, neurofeedback approaches necessitate active participation, conceivably enhancing the experience of self-efficacy. So far, studies with PTSD patients largely applied NF based on electroencephalography (EEG) recordings (3, 22–24). EEG-NF led to increased relaxation and reduced symptom severity [e.g., (23, 24)]. Furthermore, EEG-NF training shifted amygdala connectivity from areas associated with fear processing toward prefrontal control regions (3). While EEG-NF has a high temporal resolution, it lacks spatial precision. Recently, a high spatial resolution technique (rt-fMRI NF) was applied in PTSD patients. A feasibility study including three patients investigated down-regulation of amygdala activation (25). Two patients showed a clinically meaningful reduction in perceived symptom severity and a normalization of resting-state functional connectivity after the training. However, no amygdala down regulation was observed. Another rt-fMRI NF study targeted emotion regulation in patients with PTSD (26). The authors report successful down regulation of bilateral amygdala activation during NF-training that was also evident in a transfer task without feedback. Despite its importance for the psychopathology of PTSD, no study investigated ACC regulation in this patient cohort. Rt-fMRI studies in healthy individuals showed successful ACC regulation (18, 27–31). For example, pain coping during NF has been associated with activation in the ACC (32), pointing toward an important role of this structure in self-regulation in the context of aversive stimuli.

In addition to emotion regulation, the ACC has been shown to be involved in a wide range of cognitive functions, such as error detection, performance monitoring, attention, and goal-directed behavior (33–35). Sitaram et al. (36) discuss a general role of the ACC in neurofeedback studies centered at reward processing. Lawrence et al. (37) support the notion of a key role of the ACC for reward-based learning in neurofeedback paradigms. Recently, the diversity of putative roles of the ACC has been attributed to a single function: estimation of the expected value of control (35). The authors argue that the dorsal ACC determines the allocation of cognitive control by integrating information regarding the expected outcome of the control processes and their costs in terms of cognitive effort and the amount of control that is needed. In this vein, a deficit in ACC function, i.e., control allocation, may cause severe impairments in a wide range of cognitive functions. So far, no explicit strategy could be identified that consistently led to increases in ACC activation without relying on external feedback mechanisms. As a consequence, the ACC is well suited as a region of interest (ROI) for rt-fMRI NF.

The current study investigated self-regulation of the ACC in PTSD patients and matched controls. The implemented paradigm for ACC regulation is well-established, robust and has no known adverse side effects in healthy and patient populations (18, 28, 38). Here, we examined neural substrates of self-regulation in PTSD. We hypothesized that both, healthy individuals and patients with PTSD, can learn self-regulation of the ACC by means of rt-fMRI NF. We expected that learned regulation transfers to conditions without feedback and enhances performance during a cognitive interference task. In the light of previous research, we expected a weaker learning effect and reduced control of the ACC in patients. Finally, we investigated associations between symptom severity and self-regulation as well as learning in PTSD.

## Materials and methods

### Participants

Nine patients diagnosed with PTSD (age 42.3 ± 14.1) participated in the study. Diagnoses were based on ICD-10 criteria and confirmed by an experienced medical specialist for PTSD. All patients were recruited at the EUREGIO Institute for Psychosomatics and Psychotraumatology (head: G.F.) and took part in the initial phase of a specialized traumatologic treatment. We selectively chose patients who suffered from PTSD after a single trauma that did not encompass prolonged or sexual traumatization to minimize unspecific effects of repeated exposure or successive affective disturbances (39, 40). Traumata resulted from armed robberies (*n* = 4), knife attacks (*n* = 2), a car accident (*n* = 1), hostage-taking (*n* = 1), or physical violence (*n* = 1). Nine healthy controls matched for gender and age were included (age 41.3 ± 13.1). Exclusion criteria were history of psychiatric disorder, acute or past major neurological disorder, use of psychoactive drugs, any contraindications for MRI (e.g., surgical procedures in victims of accidents), and experience with neurofeedback. Handedness was not an inclusion criterion; however, apart from one left handed individual in the control group, all participants were right handed. All participants gave written informed consent after being informed about the study and prior to participation. The study was approved by the Ethical Committee of the medical faculty of the RWTH Aachen University, Germany and conducted in accordance with the Code of Ethics of the World Medical Association (Declaration of Helsinki, 2008).

### Assessment

Post-traumatic symptomatology was assessed using the German version of the impact of event scale—revised (IES-R) (41). In addition to the three subscales for intrusion, avoidance, and hyperarousal, a sum score was calculated according to Maercker and Schützwohl (41). All participants completed the German version of the extended Positive and Negative Affect Scale (PANAS-X) (42) before and after the NF training on each day. Furthermore, participants indicated if they experienced control over their own brain activation and how intense the feeling of control was on a scale ranging from 0 (not at all) to 10 (very much) on each NF day. Demographic data on age, gender, and educational level (i.e., six stages according to the German educational system) were acquired from all participants.

### Experimental stimuli and procedure

Participants were trained on 3 days to control brain activation in the ACC by means of rt-fMRI NF. On each of the training days, participants completed three NF sessions. This design allowed for the investigation of neural correlates of (self-) regulation and learning in the ACC. The BOLD signal was extracted from the ROI and fed back using social cues during NF training. Additionally, all participants performed a cognitive task without NF prior to the first NF session on day 1 and after the last NF session on day 3 to allow for: (1) the examination of transfer effects of the NF and (2) the generalization of the NF to a cognitive interference task. Accordingly, transfer was measured as change in activation patterns and generalization as change in behavioral performance from the baseline session before the training to the test session after the training.

**Figure 1:**
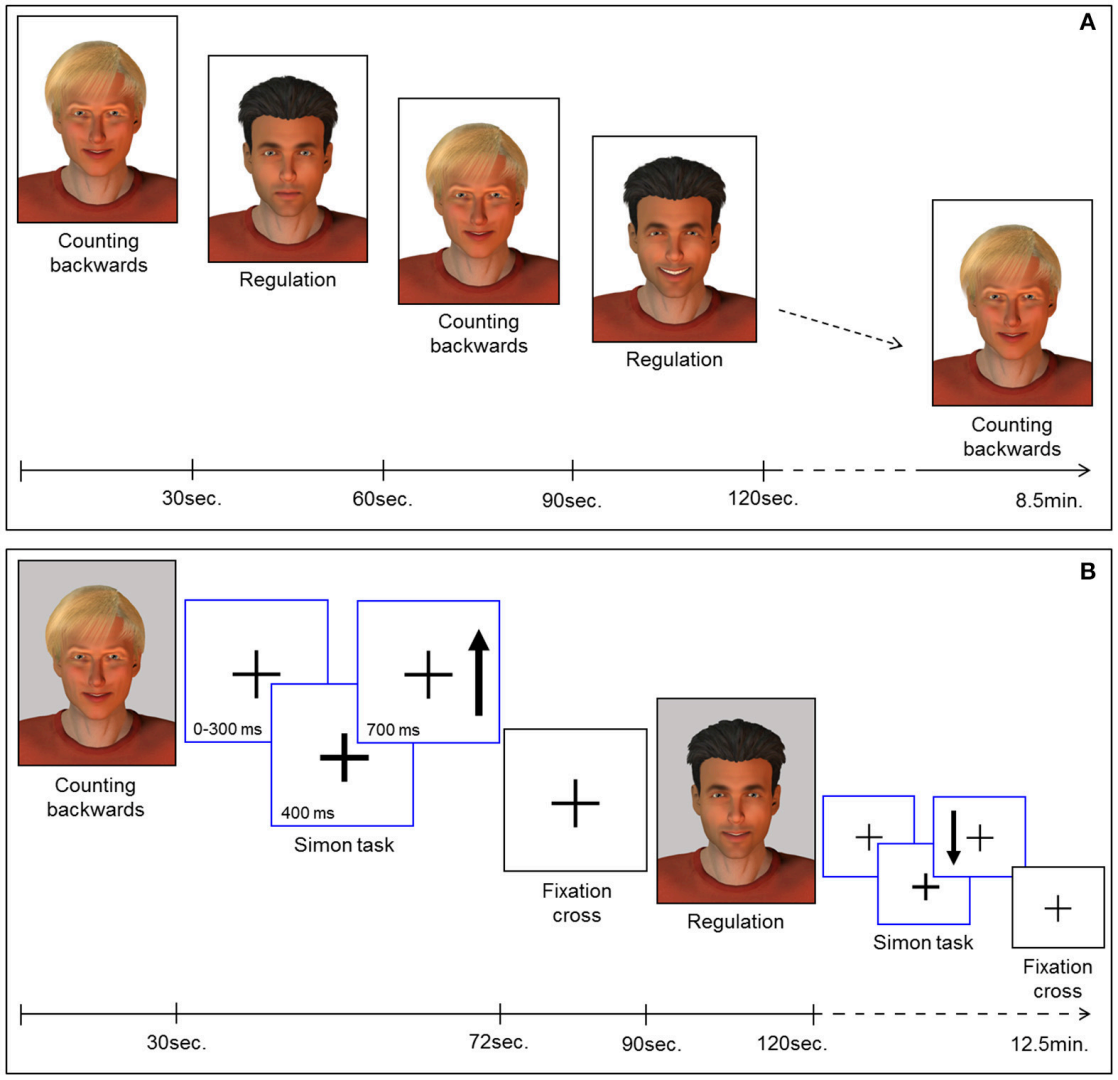
**(A)** Neurofeedback paradigm. Baseline blocks (counting backwards) and regulation blocks were presented alternately (30 s each). The expression of the avatar displayed during the regulation blocks changed with in-/or decreases of the extracted BOLD signal from the ROI. Each session comprised 9 baseline and 8 regulation blocks yielding 8.5 min total duration. **(B)** Pre-/post-test for transfer. Before and after the neurofeedback training all participants completed the task to assess transfer. In parallel to the NF task, baseline blocks and regulation blocks were presented alternately (30 s each). The expression of the avatar was static during regulation (i.e., no feedback) and baseline blocks. Each block was succeeded by a Simon task. Participants had to indicate the direction of an arrow (up vs. down) via button press (left vs. right). Subsequently a fixation cross was displayed (rest period).

Feedback of ongoing ACC activity was provided by a direct social reward. In particular, a computer-generated male face (created with Poser Pro, Smith Micro Inc.), an avatar with either black or fair-hair, was displayed. The avatar responded to an increase in ACC activity with a rewarding smile that faded with decreasing ACC activity. The facial expression was updated every repetition time (TR). The whole range of changes of the facial expression comprised 100 discreet steps ranging from neutral to a wide smile. The display of a second avatar was static and represented the baseline during which participants had to perform a counting backwards task. Counting backwards is a well-established baseline (16, 17, 31, 43, 44). Assignment of the avatars to neurofeedback or baseline condition was randomized. Each neurofeedback session comprised nine baseline and eight regulation blocks (30 s each) yielding a total duration of 8.5 min [Figure 1A; for details see (19, 29)].

All participants were asked to relax, avoid movement, and breathe evenly during the scanning sessions. They were instructed to make the avatar smile during regulation periods. In addition, they received standardized instructions on the utilization of mental strategies to learn to successfully control the ACC. In parallel to previous studies (e.g., (29)), participants were informed on mental strategies from three categories: (1) recalling positive emotional autobiographic memories, (2) imagining to perform a hobby (e.g., doing sports or practicing music), or (3) concentrating on a specific physiological perception (e.g., the temperature of their feet). These strategies served as suggestions and participants were free to follow their own approaches. Participants received detailed information about the complete neurofeedback procedure including an explanation of the delay in feedback resulting from the hemodynamic response.

### Transfer and generalization

To examine transfer effects of the neurofeedback training, participants were instructed to regulate their brain activity without feedback, both directly before (pre-test) and after (post-test) the NF sessions. An avatar was displayed in four blocks. The instructions for strategies during regulation without feedback were the same as for the neurofeedback sessions. In contrast to the NF training sessions, the facial expression of the avatar remained static during pre- and post-test sessions. A second avatar representing the baseline indicated the counting-backwards task (five blocks).

In order to investigate regulation during a cognitive interference task, participants had to perform an adapted version of the Simon task. This task was presented in eight blocks of 42 s, each displayed separately either after a baseline or a regulation block of the transfer task (Figure 1B). During the Simon task blocks, a fixation cross was presented in the middle of the screen. The appearance of an arrow was indicated 400 ms in advance by a thickening of the cross. The arrow pointed either up or down and appeared on the left or the right side of the cross. The participants were instructed to respond with button presses corresponding to the direction in which the arrows were pointing (700 ms response window). In congruent trials, button press and the position of the arrow coincided. However, in incongruent trials, a conflict occurred: the participants had to press the button on the side opposite to the position of the arrow. The average duration of a trial was 1.250 ms, enabling the display of 32 trials during each task block. Assignment of the response buttons to the direction of the arrows was counterbalanced between all participants and the events were presented in a pseudo-randomized order. After each block of the Simon task a rest period of 18 s was indicated by a fixation cross. The duration of the transfer and Simon task session was 12.5 min. Stimulus display and timing for all experimental tasks was controlled using Presentation software (Version 16.3, www.neurobs.com). Sociodemographic and behavioral data was analyzed using the Statistical Package for the Social Science (SPSS) software, version 23.

### fMRI scanning and functional data analyses

fMRI scanning was performed at a 3.0 Tesla whole body scanner (Magnetom TRIO, Siemens Medical Systems, Erlangen, Germany) with a 12-channel head coil. Echo planar imaging was used to acquire T2^*^-weighted images (EPIs) at a repetition time (TR) of 1 s (time to echo (TE) = 28 ms; 64 × 64 × 16 matrix with 3 × 3 mm^2^ resolution; 3 mm slice thickness; 0.75 mm gap). The EPIs covered 16 transverse slices in interleaved order positioned parallel to the anterior commissure—posterior commissure (AC-PC) line. The ROI was defined in each participant based on a modified anatomical template of the ACC. In particular, the ACC mask was comprised of part of the cingulate cortex such as defined in the WFU pickatlas and circumscribed by the MNI coordinates anterior to y = 0 and superior to z = 0 (18). In total, 520 volumes were obtained for each NF training run and 760 volumes for the pre- and post-test sessions, respectively.

Real-time analysis of the acquired data was performed on a separate PC using a custom Matlab toolbox for online fMRI preprocessing, analysis and feedback (for details see 18). Online motion correction was performed using spline interpolation. The BOLD signal was extracted from each voxel in the ROI during the neurofeedback training and subsequently averaged for each volume. The feedback signal was calculated as the percentage of signal change relative to the preceding baseline block with one percent signal change representing the full range of the 100 gradual frames of facial expressions of the avatar (i.e., 1% signal change resulted in the maximum smile). Low frequency drifts were removed with an exponential moving average (EMA) algorithm to improve the signal-to-noise ratio (cut-off frequency = 0.003 Hz). Furthermore, effects of outliers and high-frequency fluctuations on the signal quality were reduced by implementing a modified Kalman filter (see (17) for a detailed description of the EMA as high-pass filter and the modified Kalman filter).

Offline data analysis was conducted with SPM 12 software (Wellcome Trust Center for Neuroimaging, London, UK) implemented in Matlab R2014b (The Mathworks, MA, USA). Preprocessing of the imaging data included realignment, transformation of the acquired data into Montreal Neurological Institute (MNI) template space and spatial smoothing with an 8-mm full-width at half-maximum Gaussian kernel. The autoregressive model was fitted to the data and a 256-s high-pass filter was applied as recommended for SPM processing with a slow block design. In order to account for T1-saturation effects, the first 10 images of each scanning session were excluded. The acquired images were subjected to a general linear model (GLM) for each subject. On group level, two separate GLMs were carried out: one for the neurofeedback training sessions and one for the transfer sessions. T-maps for contrasts of interest of the second-level group analyses with regard to the regulation of brain activation were FWE-corrected at the cluster level according to *p* < 0.05 after voxel-wise thresholding (*p* < 0.001). To investigate specific effects of the regulation in the ACC, a ROI analysis was carried out (p_FWE_ < 0.05 after voxel-wise *p* < 0.001). Furthermore, the Pearson product moment correlation coefficient was calculated to investigate the association between symptom severity (such as indicated by the IES-R sum score) and the extracted BOLD signal in the ACC. A linear regression model determined the increase of regulation amplitude across session numbers to confirm the learning slope in the subjects of both groups. A group comparison allowed for the investigation of differences between groups in learning. To assess specific effects of the neurofeedback training in the ACC, the correlation between learning slopes and symptom severity such as indicated by the IES sum score was calculated. All brain coordinates are reported in MNI reference space.

## Results

### Sociodemographic data and self-report questionnaires

Patient and controls were matched for gender (8 female, 1 male each) and age [*t*_(16)_ = −0.16, *p* = 0.878]. Groups did not differ with respect to their educational level [χ^2^_(5)_ = 7.33, *p* = 0.197]. After the 3 days of training program, symptom scores were reduced yielding nominal significance for the intrusion score and on a trend-level for the avoidance and sum score (Table 1). A majority of all participants reported to have experienced self-control over their brain activation (d1: 100%; d2: 88.9%; d3: 94.4%). The level of experienced control did not differ between groups and across days such as revealed by a repeated-measures ANOVA (all *p* > 0.2). On average, participants experienced a level of moderate control on all days (Table 2). The three measurements took place within an interval of 2 weeks for all participants. The amount of days that passed between measurements did not significantly differ between groups [PTSD 6.33 ± 1.32 d, HC 7.44 ± 3.28; *t*_(16)_ = 0.94, *p* > 0.2]. The cognitive strategies that were considered successful for regulation were clustered in terms of four categories [see (38)]: (1) Music (PTSD: 11,1%, HC: 44,4%), (2) Sports (PTSD: 22.2%, HC: 66,7%), (3) Mention of other people (PTSD: 66.7%, HC: 77.8%), or (4) Others (PTSD: 88.9%, HC: 100%). Both groups predominantly relied on thoughts of other people to enhance ACC activation. The strategies in this category always involved thinking of significant others. In general, a wide range of diverse regulation strategies was utilized such as indicated by the high percentage in the category “Others.” We did not find any significant differences between groups. On a trend level, group differences emerged for the second category “Sports” [*t*_(16)_ = 2.0, *p* = 0.06].

**Table 1:** Clinical characteristics.

	**Baseline**		**Post NF**		**Comparison**	
	**Mean**	***SD***	**Mean**	***SD***	***t*_(8)_**	***p***
IES-R sum score	3.6	1.8	2.3	2.3	2.11	0.07
IES-R intrusion score	18.2	10.3	12.1	10.5	2.76	0.03
IES-R avoidance score	20.3	8.5	13.1	14.5	1.95	0.09
IES-R arousal score	17.2	11.8	10.9	10.5	1.69	0.13

*PTSD (N = 9); IES-R, impact of event scale—revised*.

**Table 2:** Perceived control and affective state.

		**Day 1**		**Day 2**		**Day 3**	
		**Mean**	***SD***	**Mean**	***SD***	**Mean**	***SD***
PC	(HC)	6	1.2	6.3	1.0	6.7	1.5
PC	(PTSD)	6.3	2.2	7.0	1.6	7.1	1.1
PA	(HC)	31.8	6.5	30.4	6.2	29.3	6.3
PA	(PTSD)	29.1	1.9	25.6	4.2	26.6	4.4
NA	(HC)	13.3	3.8	12.0	2.6	12.7	4.3
NA	(PTSD)	23.3	6.0	19.0	4.0	21.6	5.2

*PC, perceived control; PA, positive affect; NA, negative affect*.

The repeated measures ANOVA for the positive affect subscale of the extended PANAS revealed a significant main effect of days [*F*_(2, 32)_ = 4.859, *p* = 0.014; Table 2] and a significant interaction of time, i.e., pre vs. post neurofeedback sessions, and group [*F*_(1, 16)_ = 5.424, *p* = 0.033]. All other effects did not yield significance (all *p* > 0.2). For the negative affect subscale of the extended PANAS a main effect for days emerged [*F*_(2, 32)_ = 3.680, *p* = 0.036; Table 2]. All other effects remained insignificant (all *p* < 0.1). In summary, positive and negative affect changed across days and the influence of neurofeedback on positive affect such as reflected by post relative to pre measurements differed between groups.

A pooled difference score representing the change of positive affect during the neurofeedback training across all days was calculated and subjected to an independent-samples *t*-test. Groups differed significantly with patients showing on average an increase in positive affect after NF (1.5 ± 2.6) in contrast to healthy individuals [−1.4 ± 2.7; *t*_(16)_ = −2.329, *p* = 0.033]. For negative affect, change scores did not differ between groups [*t*_(16)_ = 0.717, *p* = 0.483]. Our results indicate that neurofeedback had a beneficial effect on the subjective experience of positive affect in the PTSD group relative to the control group.

Analysis of the behavioral data collected during the Simon task excluded one healthy control from the repeated measures ANOVA due to missing data. Significant main effects on reaction times were time, i.e., pre vs. post NF training, [*F*_(1, 15)_ = 20.15, *p* < 0.0001] and the Simon effect [*F*_(1, 15)_ = 18.73, *p* < 0.001]. The main effect or interactions with group did not show any significant results (all *p* > 0.2). *Post-hoc* tests revealed that all participants responded faster after neurofeedback training than before and faster during congruent compared to incongruent trials. Considering the accuracy measure, a significant interaction between time and Simon effect [*F*_(1, 15)_ = 8.76, *p* < 0.01] as well as a significant three way interaction between time, Simon effect and group [*F*_(1, 15)_ = 4.62, *p* = 0.048] emerged. The main effects for time and for the Simon effect were not significant (all *p* > 0.2).

### NF training

**Figure 2:**
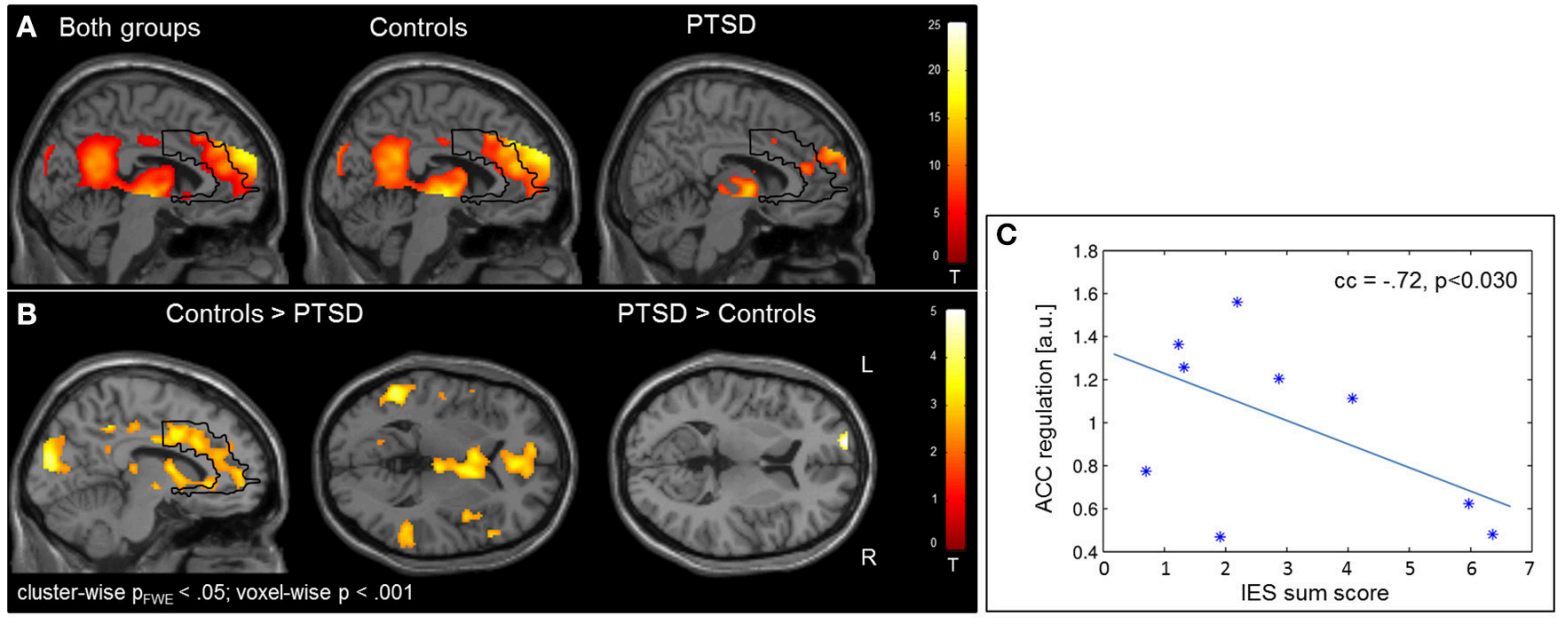
Self-regulation training. **(A)** NF of the ACC led to activation increases in multiple brain regions including the ACC, with the activation pattern being more elevated in healthy individuals. **(B)** The group comparison revealed higher activation in the visual cortex, insula, TPJ, IPL, and ACC in healthy individuals. Patients exhibited higher activation in a cluster in the left prefrontal cortex. **(C)** The IES sum score predicted ACC regulation on the first day of training.

Regulation vs. baseline condition (counting) across all days was associated with activation across multiple brain regions, encompassing prefrontal structures, insula, striatum, temporo-parietal junction (TPJ), inferior parietal lobe (IPL), lateral occipital complex, as well as posterior and anterior cingulate cortex (Figure 2A; see Table 3 for a list of activation peaks). The activation pattern encompassed the anatomically defined ROI for the ACC [(−4 44 18), T_peak_ = 11.8, p_FWE_ < 0.001]. Analyzing groups separately, controls [(−2 42 18), T = 10.84, p_FWE_ < 0.001] as well as PTSD patients [(8, 16, 40), T_peak_ = 6.97, p_FWE_ < 0.001] showed regulation of the ACC. Peak locations in both groups were similar; however, on a descriptive level statistics were lower in patients. In line with our hypothesis, a significant group difference emerged. Controls showed higher activation compared to patients in the visual cortex, frontal lobe, insula, TPJ, IPL and posterior and anterior cingulate cortex (Figure 2B). Indeed, less ACC regulation in patients was confirmed by a ROI analysis [(0 24 28), T_peak_ = 6.29, p_FWE_ < 0.001]. In the PTSD in contrast to the control group, a frontopolar/ prefrontal cluster emerged [(−14 66 8); T_peak_ = 5.09, p_FWE_ = 0.007]. Furthermore, the IES sum scores predicted ACC regulation on the first day of neurofeedback training indicating reduced ACC regulation with increasing symptom severity (cc = −0.72, *p* < 0.030; Figure 2C).

**Table 3:** Brain activation peaks.

**Cluster**	**Brain region**	**MNI coordinates**				
		**x**	**y**	**z**	**T**	***k_*E*_***
**NF** > **Baseline**						
1	Left inferior frontal gyrus	−56	20	−2	24.91	41,541
	Right superior temporal gyrus	50	−40	4	22.69	
	Left superior temporal gyrus	−48	−36	12	22.61	
	Left inferior parietal lobe	−52	−52	42	6.17	
**HC** > **PTSD**						
1	Right calcarine gyrus	20	−80	10	7.02	2,799
	Left middle occipital lobe	−18	−86	18	6.75	
	Right superior occipital gyrus	22	−78	20	6.70	
2	Superior medial frontal lobe	2	42	36	6.71	8,264
	Right frontal lobe	34	14	18	6.51	
	Anterior cingulum	0	24	28	6.29	
3	Left supramarginal lobe/left inferior parietal lobe	−64	−34	28	6.24	745
		−52	−40	28	5.63	
	Left insula	−38	−34	20	4.78	
4	Right postcentral gyrus	62	−28	20	5.13	1,120
	Right middle temporal gyrus	48	−72	10	4.69	
	Right superior temporal gyrus	56	−58	12	4.46	
**PTSD** > **HC**						
1	Frontopolar cortex	−14	66	8	5.09	79
**LINEAR REGRESSION (HC** > **PTSD)**						
1	Anterior cingulum	18	42	12	4.23	45
		16	40	6	3.95	
		18	44	8	3.78	
		14	36	6	3.63	
	Medial frontal gyrus	16	50	14	3.58	
		16	48	10	3.37	
2	Medial frontal gyrus	4	46	28	3.88	68
		4	44	18	3.78	

**Figure 3:**
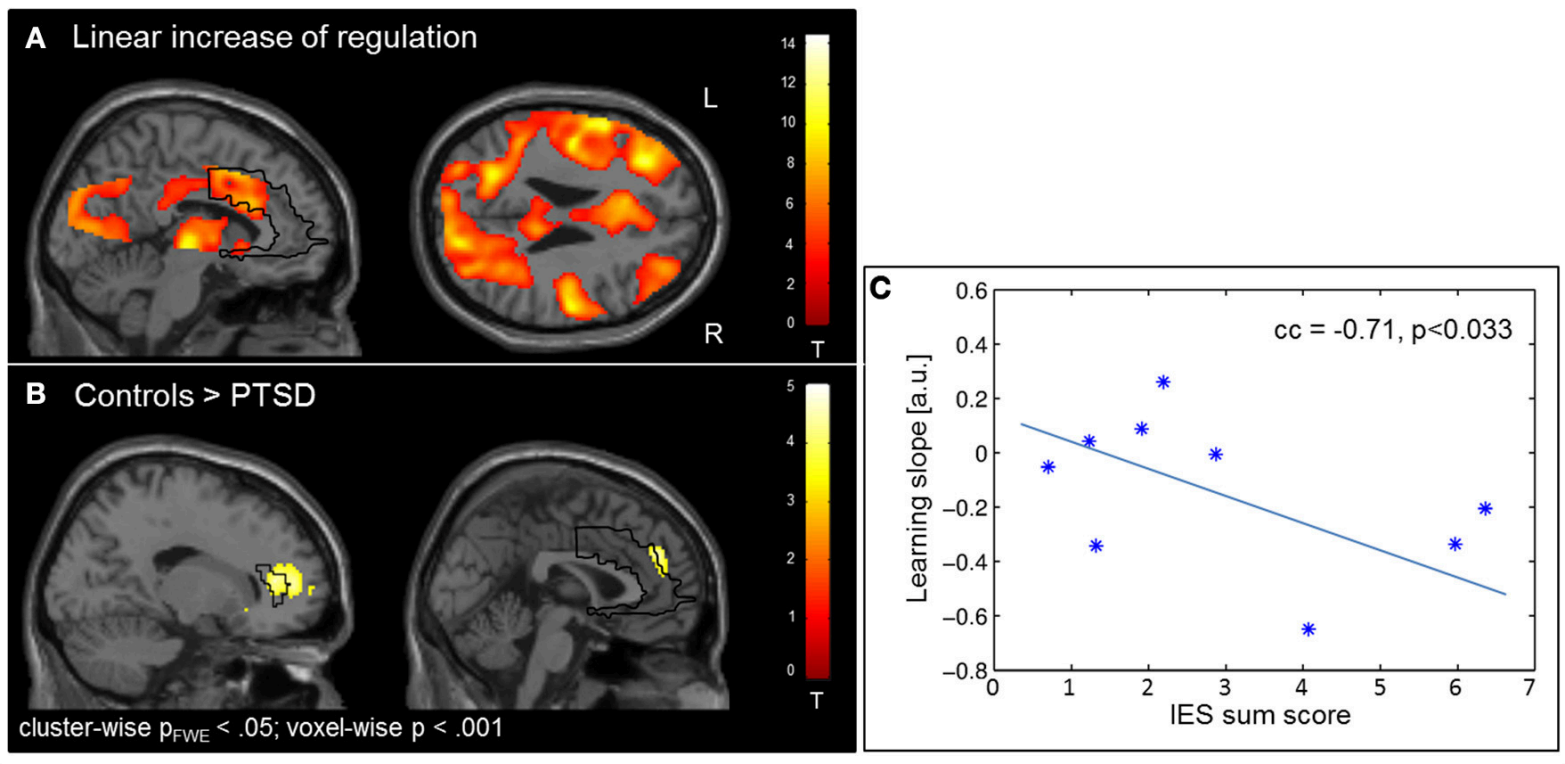
Learning. **(A)** Linear increase of regulation over sessions. Learning was observed in multiple brain regions including the ACC. **(B)** The learning rate in healthy individuals compared to patients with PTSD was particularly higher in the ACC. **(C)** Higher IES sum scores predicted less learning in the PSTD group.

The learning of control was confirmed by linear increase of regulation amplitude over sessions (Figure 3A; Table 3). The learning pattern also encompassed the ACC [(−6 22 26), T_peak_ = 8.11, p_FWE_ < 0.001] but was weaker in the PTSD compared to the control group [(12, 18, 42), T_peak_ = 4.23, p_FWE_ < 0.001; see map in Figure 3B). The learning slope in the ACC predicted IES sum scores in the patient group (cc = −0.71, *p* = 0.033; Figure 3C). In particular, a weaker learning effect was associated with higher PTSD symptom severity.

**Figure 4:**
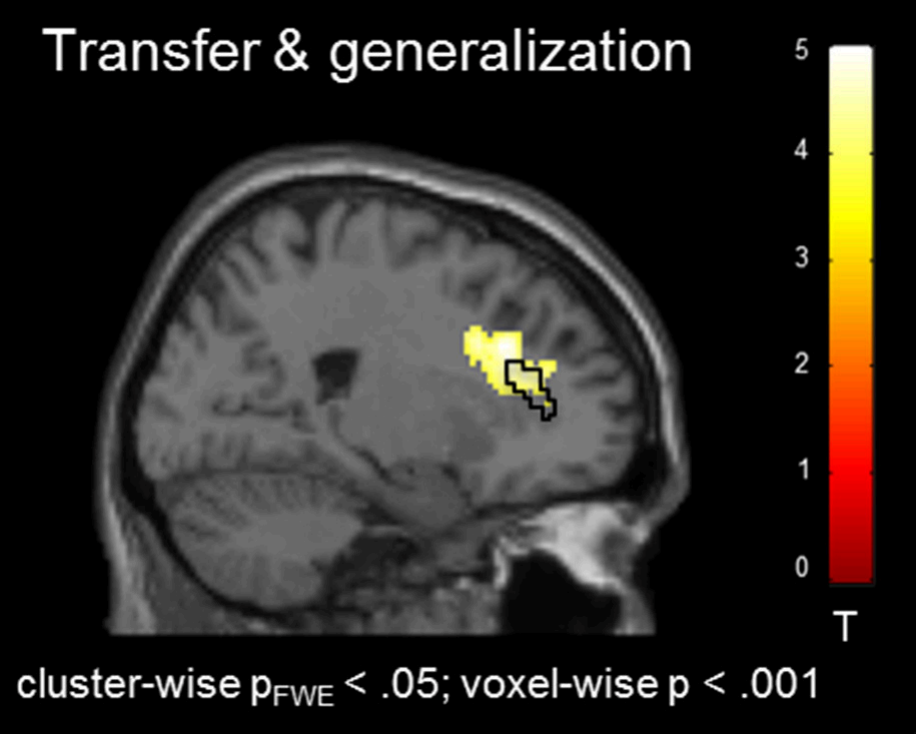
Transfer effects. Transfer of learned ACC regulation without feedback confirmed learning of self-regulation. No group differences emerged.

In the absence of a feedback signal (i.e., during the transfer task) regulation was successful over the groups, covering aspects of the ACC ROI [(22, 26, 30), T_peak_ = 4.20, p_FWE_ < 0.05; Figure 4) and confirming involvement of the ACC during regulation without feedback. At this level, no group differences emerged before or after the training at the corrected threshold.

## Discussion

Nine patients with PTSD and matched controls were trained in ACC regulation by means of rt-fMRI NF. While self-regulation of the ACC was achieved in both groups, the amplitude of regulation and learning was reduced in the PTSD group. Accordingly, one possible interpretation of the data is to hypothesize a general neural and functional impairment of ACC self-regulation in PTSD, which may contribute to the etiology. In this vein, higher IES sum scores were associated with a reduced learning effect. In apparent contrast, frontopolar areas were more active in the PTSD group during regulation presumably reflecting additional recruitment of prefrontal control regions. We showed for the first time that patients with PTSD can learn self-regulation of the ACC. Our results elucidate neural mechanisms of dysregulation in PTSD. Brain regions are identified that can be targeted in therapeutic interventions.

### Self-regulation in PTSD

From a clinical point of view, impaired self-regulation is a core feature of PTSD manifesting as impaired emotion regulation (8, 45), risk taking behavior (46), and impulsive behavior (5). Deficits in emotion regulation are closely associated with symptom severity and may constitute an underlying factor of the disorder (1). Here, we directly investigated deficits in self-regulation with rt-fMRI NF. Patients with PTSD and healthy individuals successfully learned upregulation of the ACC. In line with the beneficial effects of EEG and rt-fMRI NF that were reported in previous studies in PTSD (22, 23, 25), intrusive symptoms seemed reduced after training in patients. Furthermore, the training had a beneficial effect on the performance in a cognitive distraction task in both groups. Nicholson et al. (26) found a negative correlation between dissociative symptomatology and ACC activation during a transfer task after NF training of the amygdala. In the present study, a negative correlation between symptom severity and ACC regulation success emerged supporting the key role of this structure in the psychopathology of PTSD and self-regulation. Accordingly, in patients who experienced complex traumatization or suffer from chronic symptomatology, extended NF training may be beneficial to enhance ACC regulation. The previous rt-fMRI NF studies in patients with PTSD did not include a control group. As a consequence, no implications about self-regulation and learning in patients with PTSD relative to healthy individuals can be made. The present study extends findings by showing that regulation is reduced in patients with PTSD compared to healthy individuals presumably reflecting a general deficit in self-regulation.

Social reinforcement as compared to standard (i.e., moving bar) feedback has previously been shown to lead to improved learning of self-regulation (28). Mathiak et al. (28) suggest that social feedback facilitates operant learning during NF because it is inherently rewarding thereby omitting the necessity of explicit task knowledge. In healthy individuals, rt-fMRI NF implementing social feedback increased activation in the ACC as well as in reward-related brain areas (28). The present study replicated these findings in the group of PTSD patients and healthy controls. Activation in the ACC and the striatum was enhanced during neurofeedback. The corpus striatum plays an important role in the processing of reward-related cues (47). Accordingly, our results extend the previous study by indicating that social cues represent adequate feedback in the context of PTSD. On the behavioral level, learning of regulation transferred to subsequent trials without feedback in both groups indicating a generalization of learned regulation to other tasks. In sum, the present study adds to the evidence that the implemented NF approach yields robust regulation effects in healthy individuals and clinical samples.

Despite the observation of self-regulation of the ACC in both groups, ACC activation was reduced in PTSD patients relative to healthy controls. An explanation for reduced ACC activation and learning in patients can be derived from the neurocircuitry model of PTSD (48). The model proposes an association between decreased activation in medial prefrontal areas, including the ACC, and deficits in the control of attention in individuals with PTSD. Following this line of interpretation, the observed deficits in learning of regulation in the patient group may result from an impaired ability to focus on the task. As a consequence, responsiveness to feedback that may act as an incentive for learning in healthy individuals may be less pronounced in PTSD. Indeed, decreased motivation and deficits in attention have been associated with the disorder (49, 50). This effect may be particularly pronounced in patients with more severe symptomatology such as indicated by the association between learning slopes in the ACC and IES sum scores in patients with PTSD in the present study. However, behavioral data in the Simon task did not significantly differ between groups suggesting that a general deficit in attention or motivation is unlikely to fully account for the observed impairment in ACC regulation. It may not be trivial to distinguish between difficulties to attend to the task and to perform the regulation. We suggest that the decreased ACC activation and reduced learning reflect an underlying impairment of self-regulation in PTSD that cannot solely be explained by deficits in attention. For example, the ACC has been implicated in reward-based learning (28, 36, 37). Accordingly, learning that relies on reward-based processes may be dysfunctional in PTSD.

Given the important role of the ACC in a wide range of cognitive tasks (35) dysfunction of this structure is likely to have a substantial negative impact on the subjective well-being of the affected individuals. Previously, symptom improvement has been associated with increased ACC activation (13). In a similar vein, we observed reduced symptom scores after NF training of the ACC. Accordingly, rt-fMRI NF may be a suitable approach to enhance ACC functioning and improve symptomatology in PTSD. To verify this assumption, a study with a larger sample is needed.

In a study of ACC regulation in patients with schizophrenia (38), patients utilized different cognitive strategies yielding control of dorsal subregions of the ACC compared to regulation of the rostral ACC in healthy individuals. Presumably, emotional deficits in schizophrenia led to the development of compensatory strategies. No such pattern emerged in our group of PTSD patients. Indeed, the entire ACC was hypoactivated and exhibited less learning. Compensatory activations were only revealed in the frontopolar area that was not encompassed by the feedback region. Therefore, frontopolar regions cannot directly contribute to signal control but may indirectly influence the ACC. Fronto-limbic networks are known to play an important role in emotion regulation (10). Previously, it has been shown that four sessions of rt-fMRI NF targeting the left lateral prefrontal cortex (LPFC) can lead to a significant reduction of activation in the amygdala in healthy individuals, presumably by increased inhibition of this structure by prefrontal control regions (51).

The finding of a frontopolar cluster in the medial prefrontal cortex, that showed higher activation during regulation in patients, was unexpected. The two-pathway model of PTSD suggests that long-term exposure to trauma may lead to a dissociative subtype of the disorder that is characterized by increased prefrontal inhibition of limbic structures [i.e., hypoarousal; (35, 48)]. In contrast, hyperarousal may result from the opposite activation pattern with prefrontal areas exerting not enough control of limbic regions. Hypo- and hyperarousal symptoms are not mutually exclusive and can be experienced within the same person. In both subtypes, a deficit in self-regulation underlies the clinical symptomatology as patients exert either increased or decreased control. Clinical consequences of increased prefrontal activation such as observed in the present study may be excessive self-regulation resulting in hypoarousal and dissociation (6, 34). This view is supported by studies indicating involvement of the frontopolar cortex in cognitive control processes (52). However, the functional role of this brain region is still poorly understood and its involvement in cognitive control has recently been discussed in the context of multi-component behavior (53).

### Outlook on therapy

Rt-fMRI NF led to an increase in ACC activation indicating the potential benefits of NF approaches in the treatment of PTSD. However, the current study design does not allow for conclusions on therapeutic effects of the NF training. We can conclude that the NF procedure was well tolerated by all participants and no unwanted side effects were reported. Furthermore, symptom scores were reduced after NF training. Most participants indicated on a subjective level that they were able to control their own brain activation, reflecting the experience of self-efficacy.

Future studies are needed to investigate whether patients can learn to increase and as a consequence normalize regulation amplitudes in the ACC. This is particularly relevant in the context of complex traumatization and chronic PTSD that are typically associated with more severe symptomatology. Higher IES sum scores were negatively correlated with ACC regulation success on the first day of training and learning in patients with PTSD suggesting a need for intense training in this patient cohort. The increased frontopolar activation during neurofeedback may reflect dissociative symptomatology or hypoarousal. Therefore, differential regulation strategies need to be studied, i.e., simultaneous up-regulation of the ACC while limbic structures or frontopolar regions are downregulated (54, 55). Our findings yield interesting new perspectives for future studies investigating clinical interventions for PTSD.

### Limitations

The sample size in the present study was small. We included only patients with PTSD caused by a single trauma to avoid confounds due to a more heterogeneous symptomatology associated with complex and prolonged traumatization (40). Despite the small sample, effects of regulation and learning were detected in the present study. Possibly due to the inclusion of nine patients only, reduction in symptom scores was not significant for all subscales after NF training. However, the change in intrusive symptom scores was nominal significant and symptoms were less pronounced on a descriptive level after the training. In order to determine the effect of rt-fMRI NF on clinical variables, future studies should be randomized and double-blind. Based on the study design, we cannot exclude the possibility that beneficial effects of the NF stem from factors unrelated to the training, such as placebo or social desirability effects. However, the increase in ACC activation during regulation as well as the observed learning in both groups renders explanations solely based on social desirability unlikely.

As a further limitation, we did not implement a control condition such as sham-feedback. Indeed, artificial or sham feedback that is unrelated to regulation efforts may cause feelings of frustration. In clinical samples, advantages and disadvantages of the implementation of sham feedback—i.e., disenabling learning of self-regulation—have to be carefully considered. In healthy individuals, social feedback of the ACC has been shown to be superior to standard feedback (28) indicating its usefulness. However, the absence of a sham group in the present study limits the conclusions that can be drawn. To control for unspecific effects of the NF or attention deficits in future studies, bidirectional or sham feedback can be implemented to control for specificity.

Future research has to examine differences in regulation and predictors of treatment success in patients with PTSD compared to trauma exposed individuals without subsequent psychopathology. Findings of a recent meta-analysis suggest that comparison between these groups enables us to differentiate between global trauma induced changes and disease specific factors (7). This in turn may contribute to our understanding of resilience. In a next step, differences between patients with single and complex traumatization should be compared. This is of particular relevance since the learning slope in the ACC was negatively correlated with symptom severity indicating that patients with more severe symptoms exhibited reduced learning. Future research should investigate whether prolonged NF training can overcome differences in regulation and lead to a normalization in ACC hypoactivity in all patients. Finally, we did not differentiate between subregions of the ACC in the present study. Due to the functional heterogeneity of this region differential contributions of subregions such as dorsal and rostral parts of the ACC have to be examined more closely (7).

## Conclusion

To our knowledge this is the first study directly investigating ACC self-regulation in patients with PTSD. Regulation was achieved in the PTSD and control group, however, regulation amplitude and learning was reduced in patients. Our study confirms previous correlational research linking the psychopathology of PTSD to aberrant activation patterns in the ACC. We suggest that ACC NF training in PTSD may improve symptomatology driven by enhanced self-regulation. Suitable training programs including strategies such as neurofeedback and psychotherapy are needed.

## Author contributions

JZ wrote the manuscript. KM, JZ, and GF interpreted the findings. KM, JZ, and EP analyzed the data. KM, GF, and KM designed the study. EP and AK collected the data and MZ helped acquiring the data and setting up the study for the fMRI scanner. All authors corrected the manuscript and approved the publication.

### Conflict of interest statement

The authors declare that the research was conducted in the absence of any commercial or financial relationships that could be construed as a potential conflict of interest.
